# Exposure Assessment, Biological Monitoring, and Liver Function Tests of Operating Room Personnel Exposed to Halothane in Hamedan Hospitals, West of Iran

**Published:** 2017-11-04

**Authors:** Mohammad Hossien Bakhshaei, Abdulrahman Bahrami, Amin Mirzakhani, Hossien Mahjub, Mohammad Javad Assari

**Affiliations:** ^1^ Department of Cardiovascular Anesthesiology, School of Medicine, Hamadan University of Medical Sciences, Hamadan, Iran; ^2^ Center of Excellence for Occupational Health, Occupational Health and Safety Research Center, School of Public Health, Hamadan University of Medical Sciences, Hamadan, Iran; ^3^ Center of Research in Health Sciences, Department of Biostatistics, School of Public Health, Hamadan University of Medical Sciences, Hamadan, Iran

**Keywords:** Halothane, Aspartate Aminotransferase, Alanine Aminotransferase, Bromides, Anesthesia

## Abstract

**Background:** Occupational exposure to halogenated hydrocarbons has been associated with halothane hepatitis, an increase of liver enzymes, and congenital malformations. The objectives of this study were to investigate whether bromide, a urinary metabolite of halothane, could be used as a biological marker of exposure to this anesthetic gas and assessment of associated exposure to halothane with any significant changes in conventional parameters of liver function (serum aminotransferase activities).

**Study design:** A cross-sectional study.

**Methods:** Seventy-five anesthesiologists, anesthesia nurses, operating room nurses, and surgeons (exposed group) and 75 matched unexposed individuals (reference group) were selected randomly from two public hospitals in Hamadan City, west of Iran. Atmospheric concentrations of halothane in the breathing zone of the exposed subjects and urinary bromide levels were measured by headspace gas chromatography. Similarly, serum activities of alanine aminotransferase (ALT) and aspartate aminotransferase (AST) were measured by the enzymatic method using an automatic Prestige instrument.

**Results:** Mean atmospheric concentrations of halothane and urinary bromide levels for exposed subjects were 1.49 ±1.36 ppm and 0.83 ±0.29 mM, respectively. A relatively good correlation was found between exposure to halothane and urinary bromide levels (r=0.38). The chi-squared test results showed that the proportions of the subjects with abnormal ALT and AST among the women exposed were significantly higher than those of reference individuals (P<0.05).

**Conclusions:** Urinary bromide can be used as a potential biomarker of exposure to halothane, although additional studies are necessary to further validate these initial findings.

## Introduction

Halothane is widely used for induction and maintenance of general anesthesia. However, halothane affects the liver and kidney of exposed people and chronic inhalation exposure to excessive concentrations may lead to complications such as halothane hepatitis, elevated liver enzymes, and irreversible abnormalities^1-5^. Halothane is the only inhalational anesthetic containing bromine and causes a rare but often fatal fulminant hepatic necrosis (i.e., halothane hepatitis) resulting ultimately from oxidative halothane metabolism^6^. Emission of the anesthetic gases depends on various factors, including poor ventilation, lack of anesthetic gas cleaning systems, and poor performance during anesthesia.

Under sufficient oxygen tensions, halothane undergoes P450-catalyzed oxidation to trifluoroacetic acid (TFA), chloride, with a concomitant loss of bromine. This unstable intermediate undergoes further reactions, including hydrolysis to yield the nontoxic metabolite TFA, binding to phospholipids, and acetylation of tissue proteins to form the TFA-protein adducts ^6-8^. These protein neo-antigens stimulate an immune reaction that mediates severe hepatic necrosis. Reductive halothane metabolism generates a free radical that can eliminate bromide to form 2-chloro1,1,1-trifluoroethane (CTE), initiate lipid peroxidation, or covalently bind to phospholipids and proteins or undergo a second reduction to another free radical that eliminates fluoride to produce 2-chloro-l,l-di- fluoroethylene (CDE). The stable volatile products of halothane reduction are CTE and CDE and the stable soluble metabolites are fluoride and bromide. Aminotransferase is the most crucial and most widely used diagnostic liver enzyme that includes aspartate aminotransferase (AST) and alanine aminotransferase (ALT). SGOT or AST is naturally found in a variety of tissues, including liver, heart, muscle, kidney, and brain^6,7^. This enzyme is released into the bloodstream when a tissue is damaged ^6,7^. Maximum value of SGPT or ALT is naturally found in the liver, in contrast to AST.

Recently, new methods for the analysis of halothane or halogenated hydrocarbons in ambient air have been proposed^9-13^, and many studies have been conducted throughout the world to evaluate occupational exposure to halothane^5,14,16^. In addition, increasing bromide concentrations in the plasma and urine of some laboratory animals are reported^17-18^. A few studies have suggested the analysis of bromides in urine for evaluation to halothane, but, to the best of our knowledge, no established biological exposure index exists for halothane exposure, and little information exists concerning whether bromide, a urinary metabolite of this compound, could be used as a biological marker of exposure.

In this study, for the assessment of bromide as a biological index, the exposure of the operating room staff to halothane via inhalation was measured, and urinary bromide was analyzed at the end of the same work shift. Besides, in order to associate halothane inhalation with liver function, conventional parameters, including ALT and AST, were determined in different exposure groups.

## Methods

This study was conducted in two hospitals (Besat and Ekbatan) in Hamadan, west of Iran at 2010-2011 and the samples were collected from personnel after their operating room exposure, including anesthesia nurses, operating room nurses, anesthesiologists, and surgeons .

A total of 75 samples was obtained from exposed subjects during an operation and their urine and blood samples were collected at the end of the work shift. To remove the intervening factors, 75 healthy people as the control group were selected from the staff in other parts of the same hospitals and their urine and blood samples were also analyzed. The control group was matched with the study group based on age and smoking status and had no exposure to halothane.

Each subject gave written informed consent and the Ethics Committee of the study approved the study.

Two activated charcoal tubes (SKC, Coconut Charcoal, 20/40 mesh, 50/100 mg)^19^ in series connected to a personal sampling pump (SKC, USA) with flow of 100 ml/min were used to obtain air samples during a work shift. The charcoal tubes were attached to match subjects as closely as possible to the breathing zone for sampling from halothane. Samples were extracted with carbon disulphide^19^ and analyzed by gas chromatography (Model 4600, Unicam Company, England) equipped to flame ionization detector and a packed column (1 × 1.5m × 4 mm id with 10% peg 20 M on chromosorb W 100-120). The column temperature setting was programmed to 60 °C with an initial time of 1 min and increased at a rate of 4 °C/min to 120 °C.

To determine the urinary bromide, the urine of exposed and control groups was collected in a container at the end of each work shift. Samples were refrigerated immediately and kept frozen until analysis. To prepare urine samples, first, 0.7 ml of the sample was poured into in 10 ml septum vial, then 3.5 ml sulfuric acid and 0.7 ml of dimethyl sulfate were added and the mixture was placed in a sealed vial for 20 min at 60 °C and again for 20 min at 30 °C and, at the end, 1 ml of headspace gas from the vial was injected into the gas chromatography column (1 × 1.5 m × 4 mm id Glass column packed with Porapak Q 80-100). The initial temperature of the column was held at 110 °C for 2 min and increased to 160 °C at a rate of 4 °C per min; then the value of methyl was determined and compared to the standard curve ^20^.

To determine the parameters of the hepatic index, AST and ALT, 5 cc of subjects’ blood were collected in the exposed and control groups at the end of each work shift and transferred to the laboratory in special containers. Samples were centrifuged and analyzed in a special laboratory with the Auto Prestige Instrument Analyzer (Olympus AU640, PA, USA).

The data were analyzed by using SPSS (Chicago, IL, USA). Comparison among the mean of halothane, urinary bromide, and blood enzyme in personal samples and the control group was performed by one-way analysis of variance (ANOVA) and normality of data was confirmed by the Kolmogorov-Smirnov test. An independent t-test was used to compare values between male and female participants. The Fisher-exact test also was used to compare the proportion of abnormal blood enzyme in exposure and control groups. Logistic regression analyses was used for adjusting the important confounders such as age, sex, job duration in exposure group for abnormally in liver enzymes.

## Results

The mean concentration of halothane in the breathing zone and the biological monitoring of ALT, AST, and urinary bromide of the subjects are shown in Table 1. The results indicated a significant difference between halothane concentration in the occupational breathing zone of surgeons and other exposure groups and also anesthesia nurses and anesthesiologists (*P*= 0.042). The ANOVA statistical test showed no significant difference between the urinary bromide in the different occupations of the exposure group. The control group had no indications for urinary bromide. The statistical test also showed no significant difference among the occupational groups for concentration of liver index AST and ALT. Finally the correlation coefficient for the halothane concentration and bromide quantitative index‏ was 0.38.

**Table 1:** Results of halothane in ambient air, liver enzyme and urinary bromide in different occupations and control group

**Parameters**	**Anesthesia nurses ** **n=25 **	**Operating room nurses** **n=26**	**Anesthesiologists** **n=12**	**Surgeons** **n=12**	**Total** **n=75**	**Control group** **n=75**
Halothane in air (ppm)
X ±SD	1.58 ±1.22	1.93 ±1.83	1.38 ±0.49	0.54 ±0.14	1.49 ±1.36	0
Range	0.40 to 8.84	0.31to 4.98	0.73 to 2.14	0.22 to 0.83	0.22 to 8.84	0
Urinary bromide (mM)
X ±SD	0.83 ±0.22	0.96 ± 0.37	0.77 ±0.28	0.74 ±0.24	0.83 ±0.29	0
Range	0.42 to 1.64	0.42 to 1.35	0.54 to 1.37	0.42 to 1.29	0.42 to 1.64	0
Alanine Aminotransferase (IU/L)
X ±SD	20.32 ±6.77	22.07 ±7.52	21.41 ±4.64	20.16 ±6.91	21.37 ±6.34	18.29 ±5.37
Range	12 to 36	12 to 43	13 to 30	12 to 32	12 to 43	13 to 31
Aspartate Aminotransferase (IU/L)
X ±SD	27.56 ±4.58	28.07 ±6.25	30.41 ±6.78	25.33 ±3.75	28.12 ±4.21	25.33 ±5.10
Range	21 to 38	18 to 40	18 to 38	21 to 33	18 to 40	20 to 34

Table 2 shows the mean of AST and ALT in the exposed and control groups by gender. The results revealed a significant difference between the mean of AST and ALT in exposed female and control group (*P*=0.003) but Fisher's-Exact test showed no significant difference for ratio of abnormal AST and ALT in the whole exposed and control groups. To control of age, sex and job duration on abnormal liver enzymes the Logistic regression was used. The odds ratio for age, job duration and sex were 1.89, 0.99 and 1.09 respectively (*P*>0.05).

**Table 2:** Results of liver enzyme (ALT, AST) in different genders in exposure and control groups

**Biological index**	**Mean ±SD **	***P*** ** value**
ALT in female (IU/L) n=32		0.008
Control group	15.74 ±4.06	
Exposure group	19.62 ±6.82	
AST in female (IU/L) n=32		0.005
Control group	23.35 ±3.12	
Exposure group	27.50 ±4.82	
ALT in male (IU/L) n=43		0.113
Control group	20.09 ±5.50	
Exposure group	22.16 ±6.48	
AST in male (IU/L) n=43		0.232
Control group	26.54 ±5.80	
Exposure group	28.09 ±6.12	

The AST index in two women from the exposed group exceeds the normal limit; one of them is an anesthesia technician and other an operating room technician. Furthermore, the ALT index exceeds the normal limit for one of the anesthesia technicians, one of the operating room nurses, and two male surgeons.

## Discussion

The results of the statistical tests showed that there was a positive correlation ‏)R=0.38) between the value of inhalation halothane‏ and urinary bromide. The value of the correlation coefficient is concern to metabolism process of inhalation halothane. About 60% to 80% of inhaled halothane, depending on the activity at the initial time, is excreted through exhalation. Only 20% to 40% of halothane is released into the biotransformation cycle^7,8^, which, depending on the individual, may affect the liver, affect physiological characteristics, and increase the amount liver enzyme in the urine and cause to produce methyl bromide in urine.

The lack of bromide in the urine in samples of the control group and the emergence of this index in the urine of the exposure group showed that exposure to halothane causes the appearance of this index in the exposure group. A significant difference existed between the values of exposure to halothane concentration among occupational groups. The inter-group difference of halothane exposure in the exposure groups may be due to the difference in mean of working hours and concentrations of halothane exposure.

Our results showed significant difference for AST and ALT in exposed female and control group but no significant difference for male exposed occupations. It may concern to effects of halothane on enzymes level on women people. The role of gonadal hormones in regulating structure and function of nearly every tissue and organ in the mammalian body, causing gender differences in a variety of characteristics is reported ^21-23^. Amount of direct bilirubin, AST and albumin levels in women are low compared to men^23^ and it may more get effects than men people in front of some external factors.

The American Conference of Industrial Hygiene Association (ACGIH) has not suggested any biological index for exposure to halothane ^24^. A positive correlation between the halothane in ambient air and urinary bromide suggests that this compound can be used as an index when exposed to halothane.

The maximum value of the methyl bromide index was 1.64 mM, observed in one of the anesthesia nurses who was accidentally exposed to the highest level of halothane (8.84 ppm). This sample may confirm that increased exposure to halothane increases the urinary bromide of exposed people.

To evaluate human exposures to halothane, the analysis of biomarkers, such as bromide in the urine is more convenient than measuring air concentrations and inhalational exposures to anesthetic compounds, including halothane. The Occupational Safety and Health Administration’s (OSHA’s) method 103 is the method of choice, and used extensively for exposure assessment of halothane (OSHA 2010)^19^. Despite all of the positive points associated with this method, it has some critical deficiencies in real-world applications. For example, the tasks undertaken by people in these environments are very complicated and demanding, making it cumbersome and inconvenient for surgeons and technicians to wear a full sampling train (pump, tubing, and adsorbent tubes).

For biological monitoring, various studies have proposed occupational exposure to halothane based on measurement of concentrations of the biotransformation products in blood, halothane in alveolar air and urine, and inorganic urinary bromide^20, 25-26^,. Besides, some new methods were recommended for analysis of anesthesia in air, urine and blood using of solid phase micro extraction (SPME) and needle trap devices. The microextraction methods have high sensitivity with new sensitive adsorbant. SPME has some drawbacks, such as fiber fragility, which can cause fiber breakage from mechanical stress during the sample taking and sample delivery stages, and limited sorption capacity. Most of the research has been associated with the development of analytical methods and the measurement of bromide in the urine of laboratory animals. Hankins et al. developed a method in which they used high-performance liquid chromatography-ion chromatography (HPLC-IC) with suppressed conductivity detection for the simultaneous detection of both trifluoroacetic acid and bromide in plasma and urine^27^. In another study, the increase of fluoride and bromide concentrations in plasma was observed when enflurane was used, and the increase in the fluoride concentration in the plasma could not be distinguished from normal individual variations^28^. However, the concentration of bromide in the plasma increased significantly when halothane was used. Increased concentrations of bromide were reported in the plasma of pregnant mice exposed to halothane at various concentrations for 1 hour^29^.

In our study, we limited the exposure to halothane to low concentrations, and we were unable to determine the limit value for bromide in the urine associated with inhaled halothane. Results of this study suggest that halothane should be replaced by other new and less risky anesthetics, using modern and updated anesthetic machines and staff training to achieve desired outcomes for anesthesia machines; also, operating room ventilation systems should be improved.

## Conclusions

Due to the correlation coefficient between the concentration of halothane and the quantitative index of methyl bromide, this index can be used to evaluate occupational exposures to halothane. Besides, exposures to sub-TLV levels of halothane were not associated with liver dysfunction or pathology. However, more studies with high exposure are need to further validate these initial findings.

## Acknowledgements

This research was part of MSC Thesis at Hamadan University of Medical Sciences, Hamadan, Iran. We thank for financial support (Grant No. 48729) for this research.

## Conflict of interest statement

The authors declare that they have no conflicts of interest.

## Funding

This study was funded by the Vice-Chancellor of Research and Technology, Hamadan University of Medical Sciences, (Grant No. 48729).

## Highlights

- High level exposure to halothane may lead to complications.
- No established biological exposure index (BEI) exists for halothane exposure.
- The urinary bromide can be used as an index for evaluation of exposure to halothane.
